# Clear Cell Renal Cell Carcinoma: Rare and Atypical Metastatic Localizations—A Case Series

**DOI:** 10.1155/criu/1777980

**Published:** 2026-04-16

**Authors:** Roosvelt Tessa Djambong, Bril Laurel Follah Feudjio, Stéphane Rysselinck, Emmanuel Seront, Günter Niegisch, Christina Neppl, Alexander Müller

**Affiliations:** ^1^ Department of Internal Medicine, Hôpital du Jura, Delémont, Switzerland, h-ju.ch; ^2^ Department of Urology, Uroviva Spital Limmattal, Schlieren, Switzerland; ^3^ Department of Urology, CHU HELORA La Louvière-Site Jolimont, La Louvière, Belgium; ^4^ Department of Medical Oncology, Cliniques universitaires Saint-Luc, Brussels, Belgium, saintluc.be; ^5^ Department of Urology, Medical Faculty and University Hospital, Heinrich-Heine-University, Düsseldorf, Germany, uni-duesseldorf.de; ^6^ Center for Integrated Oncology (CIO) Düsseldorf, CIO Aachen-Bonn-Köln-Düsseldorf, Düsseldorf, Germany; ^7^ Department of Pathology, Medical Faculty and University Hospital, Heinrich-Heine-University, Düsseldorf, Germany, uni-duesseldorf.de

**Keywords:** biliary metastases, clear cell renal cell carcinoma, nasal metastases, pancreatic metastases, thyroid metastases

## Abstract

**Introduction:**

Clear cell renal cell carcinoma (ccRCC) is the most prevalent subtype of kidney cancer, characterized by its high degree of vascularity and metastatic potential. Although common metastatic sites include the lungs, lymph nodes, bones, liver, adrenal glands, and brain, rare localizations, such as those observed in the pancreas, gallbladder, thyroid gland, and nasal cavities, pose significant diagnostic and therapeutic challenges.

**Case presentation:**

The following report details four cases of ccRCC with atypical metastatic localizations: A nasal metastasis in one patient, a thyroid metastasis in one patient, a gallbladder metastasis in one patient, and pancreatic localizations in all four patients. These metastases, frequently asymptomatic, contributed to delays in diagnosis. The therapeutic management of these cases was individualized, incorporating surveillance, surgical intervention, radiotherapy, or immunotherapy, depending on the patient′s clinical profile and the progression of the lesions.

**Conclusion:**

This series illustrates the diversity of metastatic presentations of ccRCC and highlights the importance of prolonged, personalized follow‐up after nephrectomy. A multidisciplinary approach remains essential for the timely detection and optimal management of rare metastatic sites.

## 1. Introduction

Clear cell renal cell carcinoma (ccRCC) accounts for approximately 75%–85% of renal cancer subtypes [1]. At the time of diagnosis, approximately 30% of patients present with metastatic disease, and additional cases may develop distant metastases after treatment of localized tumors. The most frequent sites of metastasis are the lungs (70%), followed by the lymph nodes (45%), bones (32%), liver (18%), adrenal glands (10%), and brain (8%) [2, 3]. However, metastases can occasionally occur in other organs, for instance, pancreatic metastases, which are among the most prevalent secondary malignancies of the pancreas, arising from ccRCC [4, 5]. Rare sites of metastasis also include the thyroid gland, gallbladder, and nasal cavity. These metastases are predominantly asymptomatic and are often detected years after treatment of the primary tumor. The diagnosis is of significance as it may necessitate a proactive local approach in the context of patient management.

In this study, we reported a series of four cases with uncommon sites of metastasis. The aim is to highlight the importance of recognizing these atypical localizations during long‐term follow‐up and to review current therapeutic approaches based on available literature.

## 2. Case Presentation

### 2.1. Case 1

**Figure 1:**
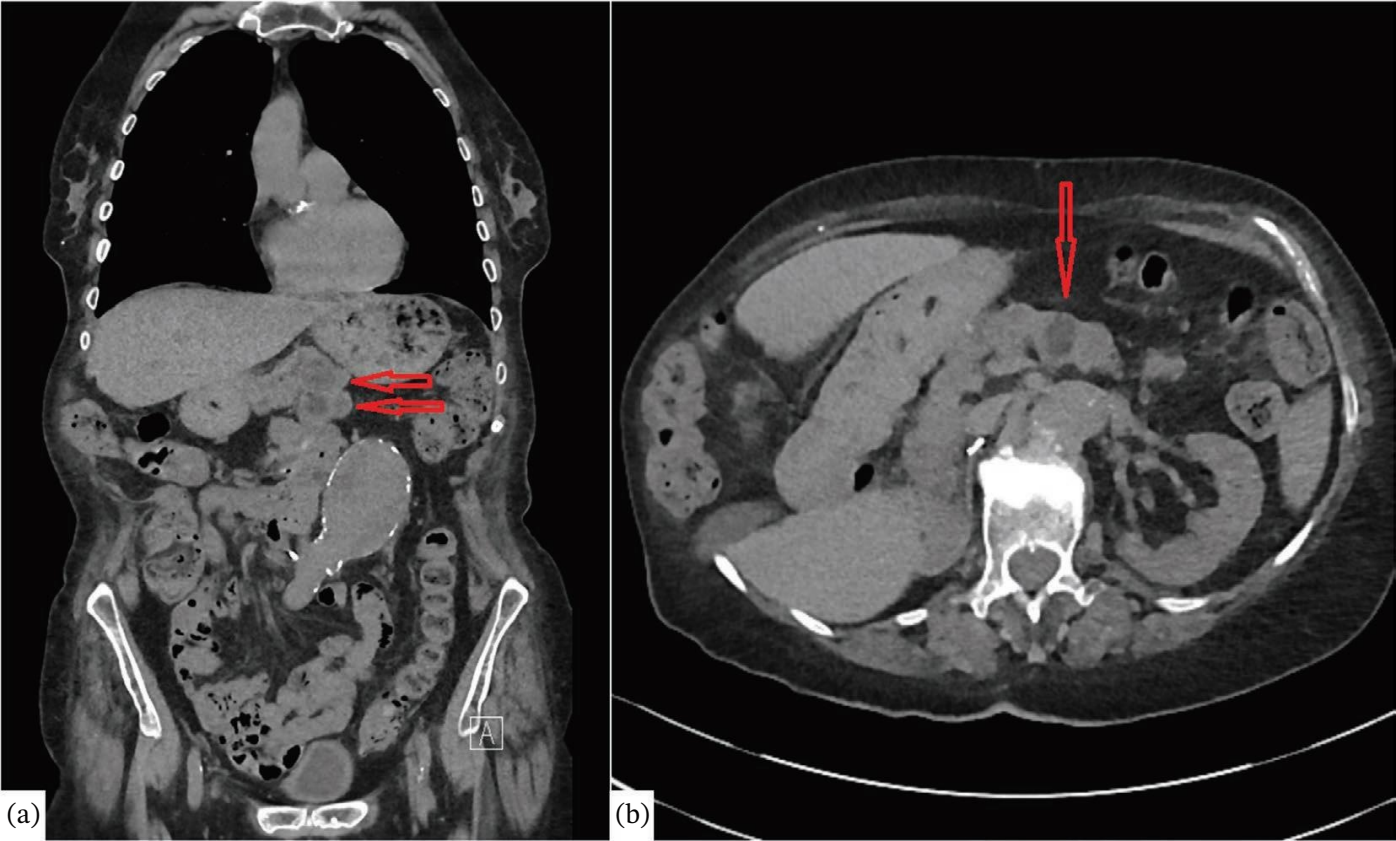
Abdominal CT scan findings consistent with pancreatic metastases (red arrow)—(a) coronal view; (b) axial view.

A 77‐year‐old female patient was referred to the urology consultation in April 2013 following the demonstration of a 10 × 8 cm tumoral lesion of the right kidney, revealed by an abdominal ultrasound performed as part of a workup for vomiting, asthenia, and weight loss (15 kg in a few days). A subsequent thoraco‐abdominopelvic CT scan confirmed the presence of a tumoral lesion, accompanied by a substantial zone of necrosis and a thrombus in the right renal vein (Figure 1). Two infracentimetric pulmonary nodules consistent with scar‐like (cicatricial) lesions but no metastases were detected. A bone scan was likewise inconspicuous.

Right radical nephrectomy was performed by lumbotomy in April 2013. Histological analysis of the surgical specimen confirmed ccRCC, with the following Tumor‐Node‐Metastasis (TNM) classification: pT3b, pN0, cM0, R0, and Fuhrman Grade II. Postoperative follow‐up was uncomplicated, and oncological monitoring was conducted in accordance with prevailing recommendations.

Three years following the surgical procedure in March 2016, during a medical examination for persistent diarrhea, a computed tomography (CT) scan of the abdomen revealed the presence of two nodules in the head of the pancreas (15 and 10 mm, respectively). Echo‐endoscopy was performed, and both lesions were biopsied. A thorough pathological examination revealed the presence of tumorous proliferation, consisting of monomorphic cells with clear cytoplasm, arranged in nests, and positive for anti‐CD10 and antivimentin immunostaining. However, the samples were negative for anti‐EMA immunostaining. The presence of these features indicated a probable metastasis of ccRCC. To complete the evaluation of the patient′s condition, an FDG‐PET scan was performed; however, it did not yield any additional information, as the lesions that were visible on the CT scan did not show increased FDG uptake.

A re‐examination of the original CT scans reveals that these pancreatic lesions were already evident at the 2‐year checkup following nephrectomy and have remained stable and asymptomatic ever since.

The patient′s case was discussed in a multidisciplinary tumor board. In consideration of the patient′s age and the asymptomatic nature of the lesions, a surveillance approach was recommended.

During follow‐up, the patient exhibited slowly growing pulmonary nodules but continued to be asymptomatic. A thoracic‐abdominalpelvic CT scan performed in October 2024 revealed progression of the pancreatic lesion localized to the body of the gland, with an impression of compression of the digestive tract, with no significant clinical impact. In view of this progression, the treatment strategy was discussed in a multidisciplinary tumor board. Considering the patient′s advanced age (88 years), frailty, and ECOG performance status of 2, combination therapy with immune checkpoint inhibitors (ICIs) and VEGFR‐TKI was considered potentially poorly tolerated. The patient was classified as intermediate risk (1 criteria; Karnofsky Performance Status 60%) according to the International Metastatic Renal Cell Carcinoma Database Consortium (IMDC) criteria. In this context, association of VEGFR‐TKI plus ICI was suggested. However, based on the angiogenic profile of the cancer (glandular metastases, interval between nephrectomy and systemic treatment start > 1 year, no hematological anomaly), we started with axitinib alone with close follow‐up. Because a response was observed on axitinib alone, we did not start pembrolizumab. The therapeutic decision was therefore individualized, taking into account the patient′s clinical condition and expected treatment tolerability. The patient demonstrated good tolerance to the treatment. A radiological assessment was conducted after 6 months (March 2025), revealing a partial response, accompanied by a substantial reduction in the volume of the pancreatic tumor (diminished from 37 to 22 mm). This development was concomitant with the elimination of presumed digestive compression. Concomitant with these observations, a reduction in the size of the pulmonary nodules was also noted on subsequent imaging. In consideration of the patient′s satisfactory tolerance levels and the encouraging radiological response observed, the decision was made to proceed with the ongoing treatment regimen. The patient is still alive as of June 2025.

### 2.2. Case 2

A 76‐year‐old female patient was referred to the general surgery department in July 2014 for evaluation of a right supraclavicular swelling that had been noted 10 days prior without any associated symptoms.

The patient′s blood analysis revealed euthyroidism, normal phosphorus and calcium levels, and negative anti‐TPO and anti‐TSI antibodies. CEA levels exhibited a marginal increase, registering at 4.9 *μ*g/L.

A subsequent ultrasound examination revealed an increase in the volume of the right thyroid lobe, estimated at +/−17 mL, with the presence of two fleshy thyroid nodules on the same side. The larger nodule, measuring 8 mL, exhibited a long axis of 3 cm, whereas the second, measuring 0.7 mL, demonstrated a long axis of 1 cm.

Analysis of a thyroid puncture showed only acellular serositis, which proved to be noncausative. A thyroid lobectomy was performed in September 2014. Histological analysis of the surgical specimen revealed that the nodule was composed of cells with abundant, clarified cytoplasm. Subsequent immunohistochemical analysis revealed that the cells within this tumoral nodule exhibited a negative reaction for both antithyroglobulin antibodies and neuroendocrine markers. However, the presence of anti‐CD10 and antivimentin antibodies was detected, suggesting a renal origin for this glandular proliferation. Consequently, the diagnosis was a metastatic clear‐cell adenocarcinoma of renal origin.

**Figure 2:**
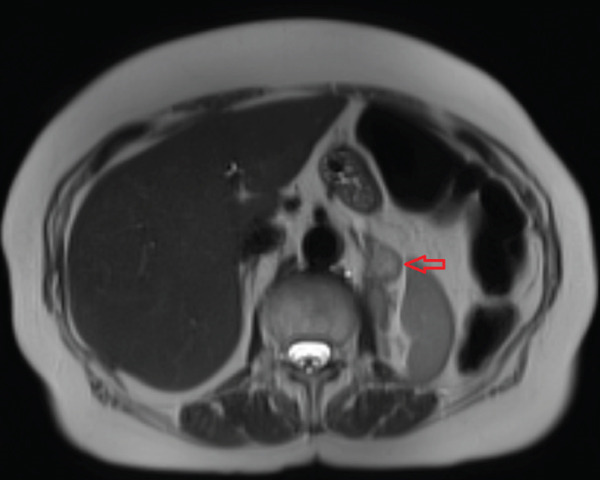
Axial abdominal MRI findings consistent with pancreatic metastases (red arrow).

A subsequent thoracoabdominal CT scan was performed, which confirmed the presence of a voluminous left renal neoplastic lesion measuring 96 × 73 × 70 mm, with tumoral invasion of the left superior polar renal vein (Figure 2). No additional metastases were identified. A bone scan and a brain MRI were conducted, revealing no distant lesions.

A laparoscopic radical left nephrectomy was performed in October 2014. Histological examination a Fuhrman I grade ccRCC, classified as pT3a pN0.

A subsequent CT scan, performed 5 months after the nephrectomy, revealed the presence of an intrapancreatic lesion that, in retrospect, was already present prior to the surgical procedure.

The patient′s case was discussed in a multidisciplinary tumor board. Given the patient′s age and the asymptomatic nature of the lesions, a surveillance approach was recommended and followed.

A subsequent CT scan conducted 17 months after the initial procedure (March 2016) revealed the presence of two fleshy, heterogeneous, hypercapturing nodular formations, with dimensions of 16 and 22 mm, respectively. The lesions, which were located in the tail of the pancreas, demonstrated stability in comparison with the examination conducted 6 months prior. However, a discernible progression was observed in relation to the preoperative examination. Concurrent MRI imaging revealed two hypervascularized lesions in the tail of the pancreas.

The patient′s case was discussed once more in a multidisciplinary consultation, but the surveillance approach was maintained.

The patient passed away at the age of 80 years old, 4 years later, from a cause unrelated to the progression of the initial renal neoplasia.

### 2.3. Case 3

**Figure 3:**
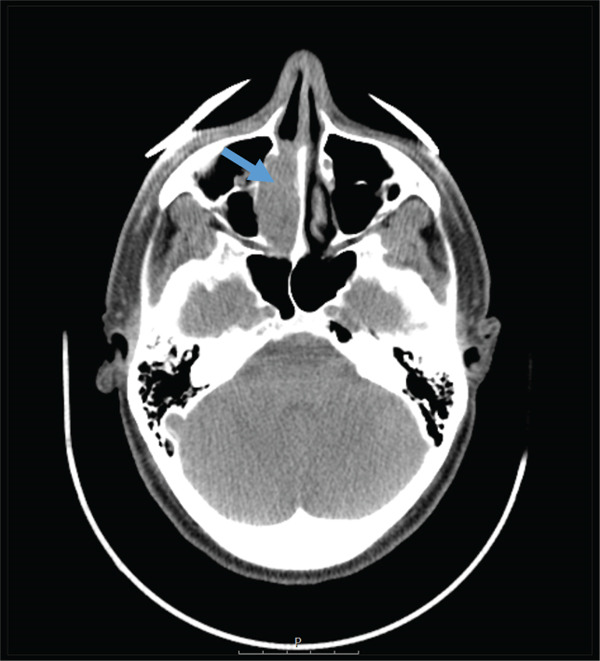
Axial CT scan of the brain findings consistent with a suspicious lesion of the papyraceous lamina with subocclusion of the nasal cavity (blue arrow).

A 46‐year‐old patient with a medical history of left total nephrectomy due to ccRCC 15 years ago (2004), as well as nasal septoplasty performed on December 4, 2019 for recurrent epistaxis, was admitted to the emergency department 2 days later for severe right‐sided epistaxis. As the initial surgical procedures were performed at another institution, detailed information regarding the surgical approaches is unfortunately unavailable. A CT‐angiogram was performed, which revealed a suspicious lesion of the papyraceous lamina with subocclusion of the nasal cavity (Figure 3). The patient underwent emergency embolization of the right maxillary artery, followed by resection of the lesion and placement of a Bellocq‐type tamponade.

**Figure 4:**
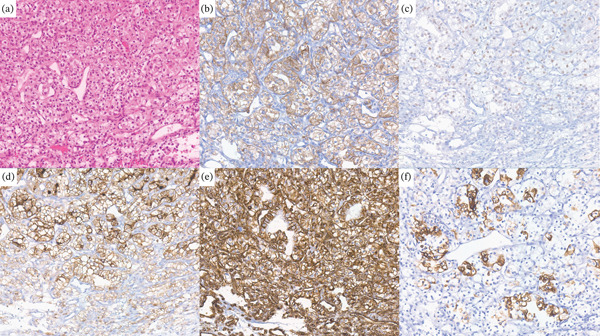
Immunohistochemical analysis of nasal tumor cells: (a) Positive for HE; (b) positive for CKAE1/3; (c) positive for PAX8; (d) positive for CD10; (e) positive for vimentin; and (f) positive for CK7.

Histological analysis revealed formations of a tumor with solid to nest‐like, and in part also trabecular, growth. The atypical cells exhibited a prominent light, in some areas slightly eosinophilic, cytoplasm and relatively small, round nuclei. In additional immunohistochemical tests, the tumor was positive for CK AE 1/3, CD10, vimentin, partially for CK7, as well as showing nuclear positivity for PAX8 (Figure 4).

An FDG‐PET CT‐scan (December 2019) revealed suspected local recurrence at the nephrectomy site, as well as cervical lymphonodal metastases. Two round, soft–tissue‐density masses (2.0 × 1.8 and 1.4 × 1.0 cm) with contact to the upper left psoas muscle, showing moderately increased FDG uptake, were detected. Further, caudal and dorsal to the former renal fossa, likely along the surgical access route, there was another multiperfused soft‐tissue mass measuring 4.2 × 2.3 × 3.6 cm with similarly moderate FDG uptake. In addition, formally nonpathologic cervical lymph nodes showing heightened FDG uptake (e.g., a 9 mm node in the right mandibular angle, a 1 cm node near the left maxillary sinus, and a right submental node in the dorsal region) were seen. A brain magnetic resonance imaging (MRI) revealed no evidence of brain metastases.

The patient′s case was discussed in a multidisciplinary tumor board. Given the patient′s relatively young age, surgical resection of the suspected lesions as well as systemic combination treatment with the tyrosine kinase inhibitor axitinib and the PD‐1 inhibitor pembrolizumab, according to the KEYNOTE‐426 trial [6], was recommended.

**Figure 5:**
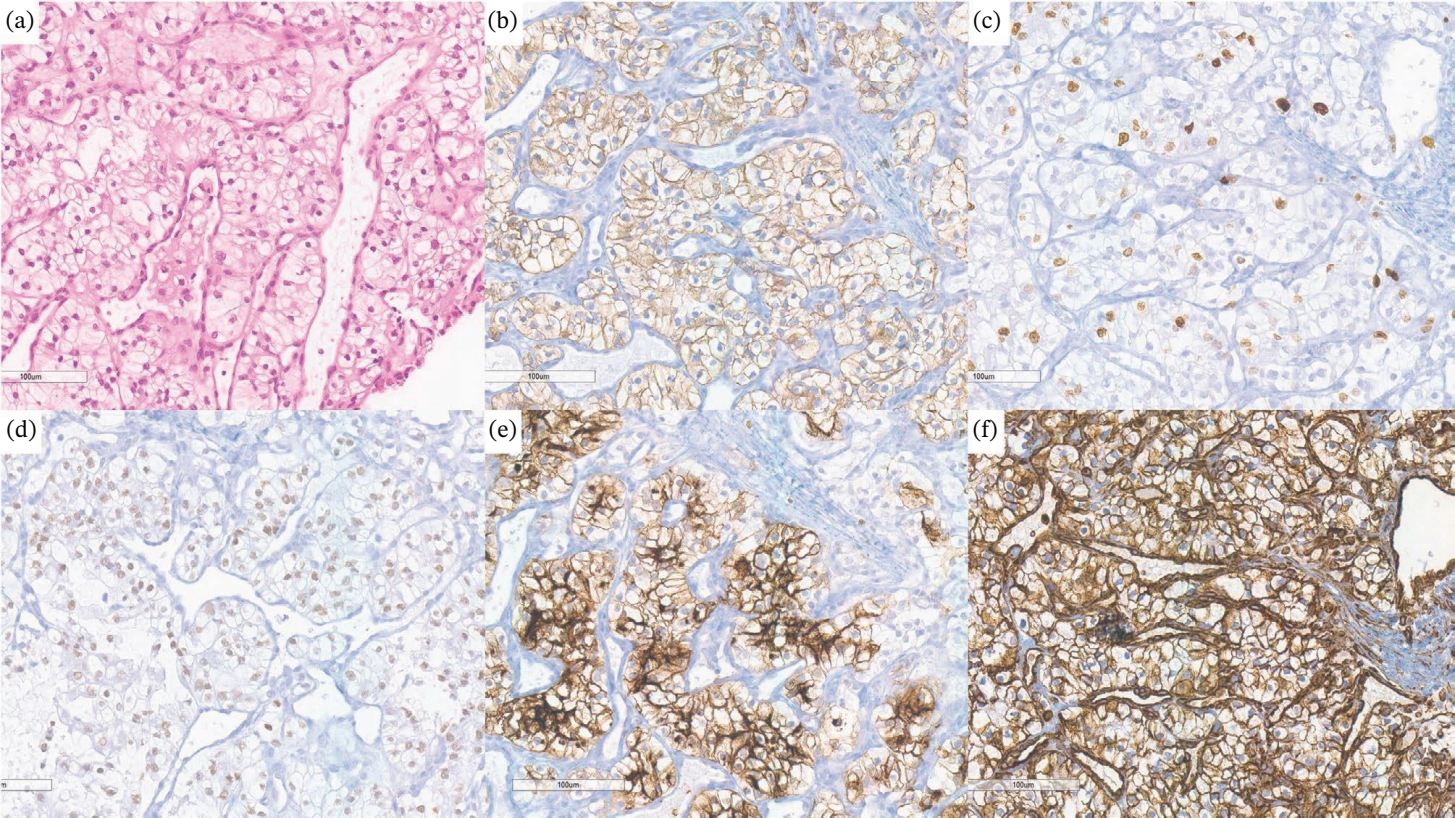
Immunohistochemical analysis of tumor cells in retroperitoneal lymph nodes: (a) Positive for HE; (b) positive for CKAE1/3; (c) positive for MIB‐1; (d) positive for PAX8; (e) positive for CD10; and (f) positive for vimentin.

The patient was admitted to the hospital one and a half months later for surgical intervention. However, a subsequent thoraco‐abdominopelvic CT scan (February 2020) revealed three new metastatic lesions upon admission: a segment three liver lesion (measuring 0.5 × 0.8 cm), two pancreatic lesions (one in the head of the pancreas, measuring 1.4 × 2.2 cm, and the other in the body of the pancreas, measuring 1.6 × 2.3 cm). The operation was subsequently canceled, and the case was discussed again in a multidisciplinary consultation. It was recommended that a biopsy be performed on one of the abdominal metastases, specifically on the psoas muscle, and that, subsequent to histological confirmation, immunotherapy be initiated with pembrolizumab and axitinib. Given the patient′s young age, surgical excision of each metastatic lesion was also recommended. A biopsy of the metastatic lesion confirmed metastasis of ccRCC. Representative photographs are shown in Figure 5.

**Figure 6:**
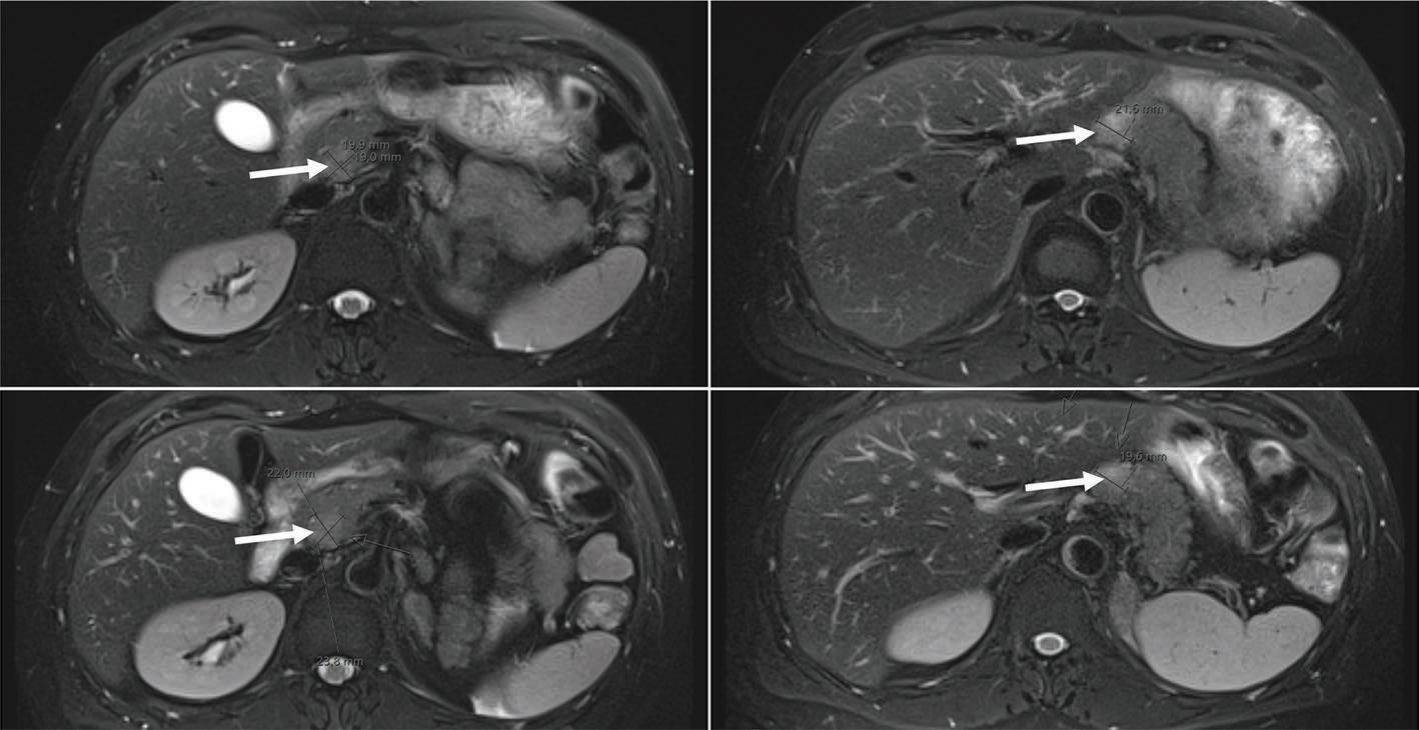
Axial abdominal MRI findings consistent with pancreatic metastases (white arrow).

In March 2020, the patient started first‐line systemic therapy with pembrolizumab and axitinib with a subsequent partial response. In December 2021, abdominal MRI showed size‐constant pancreatic metastases (Figure 6) and suspicion of at least three small liver metastases in Segment III and at the border between Segments II and IVa. In the same month, chemoembolization of the liver metastases in Segments II and III was performed. Although a change in systemic therapy would formally be indicated for newly detected liver metastasis, the multidisciplinary team (MDT) initially opted for a trial of metastasis‐directed therapy in this case. Key reasons included the oligoprogression itself and the low tumor burden at that time. According to the MDT, delaying a switch in systemic therapy until further progression was demonstrated would not have been disadvantageous for the patient. Careful consideration of potential harms and benefits with regard to both switch of systemic treatment and modality of metastasis‐directed therapy was also taken into account within the further patient history as was effectiveness of metastasis‐directed therapy in this individual case.

In December 2023, another chemoembolization was carried out on a liver metastasis in Segment VIII. Due to size progression of the liver metastasis in Segment VIII, a further chemoembolization was performed in May 2024. In June 2024, radiofrequency ablation of a new left retroperitoneal metastasis was also performed. Restaging in October 2024 showed a slight size progression of the liver metastases with otherwise stable disease; this finding was confirmed again in February 2025. In May 2025, the patient suffered a myocardial infarction, leading to a 4‐week pause of axitinib. Subsequent restaging revealed a slight hepatic progression with otherwise stable findings. In June 2025, the patient resumed treatment with axitinib and pembrolizumab. Though restaging in August 2025 showed stable disease, progression at multiple sites including, for the first time, osseous (left acetabulum) and pulmonary metastases was seen in November 2025.

Here, systemic treatment was switched to cabozantinib and radiotherapy of the osseous metastases was initiated. Restaging in January 2026 (performed early due to unspecific lumbar pain) showed a partial remission including a near complete remission of hepatic and pancreatic metastases.

Apart from minor side effects of the current therapy, the patient is in good condition and still fully employed.

### 2.4. Case 4

A 70‐year‐old female patient was found to have a 69 × 64 mm lesion in the right kidney, as revealed by abdominal CT in September 2022, which was deemed suspicious and indicative of macrohematuria. No metastases or suspicious lymph nodes were identified. Open total nephrectomy for symptomatic renal mass was performed 12 days after diagnosis. A histological evaluation revealed ccRCC, grade 2 according to the WHO/ISUP 2016 classification, graded pT2a R0.

**Figure 7:**
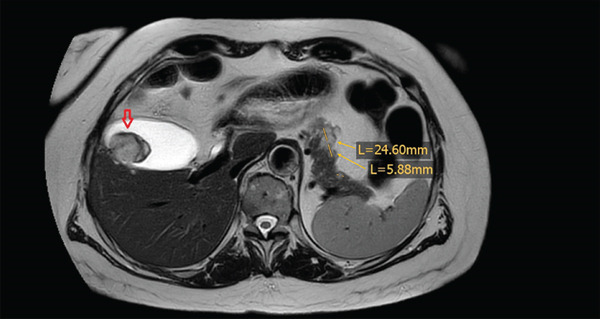
Abdominal MRI showing metastases in the gall bladder (red arrow) and pancreas (orange arrow).

A subsequent retrospective analysis of preoperative images revealed a 3‐centimeter lesion in the gallbladder and a lesion in the body of the pancreas, both of which were considered to be metastases. Subsequent MRI analysis corroborated these initial suspicions (Figure 7).

The patient′s case was reviewed during a multidisciplinary conference in December 2022, and it was determined that further intervention was necessary. Specifically, the decision was made to proceed with a cholecystectomy and biopsy of the pancreatic lesion.

**Figure 8:**
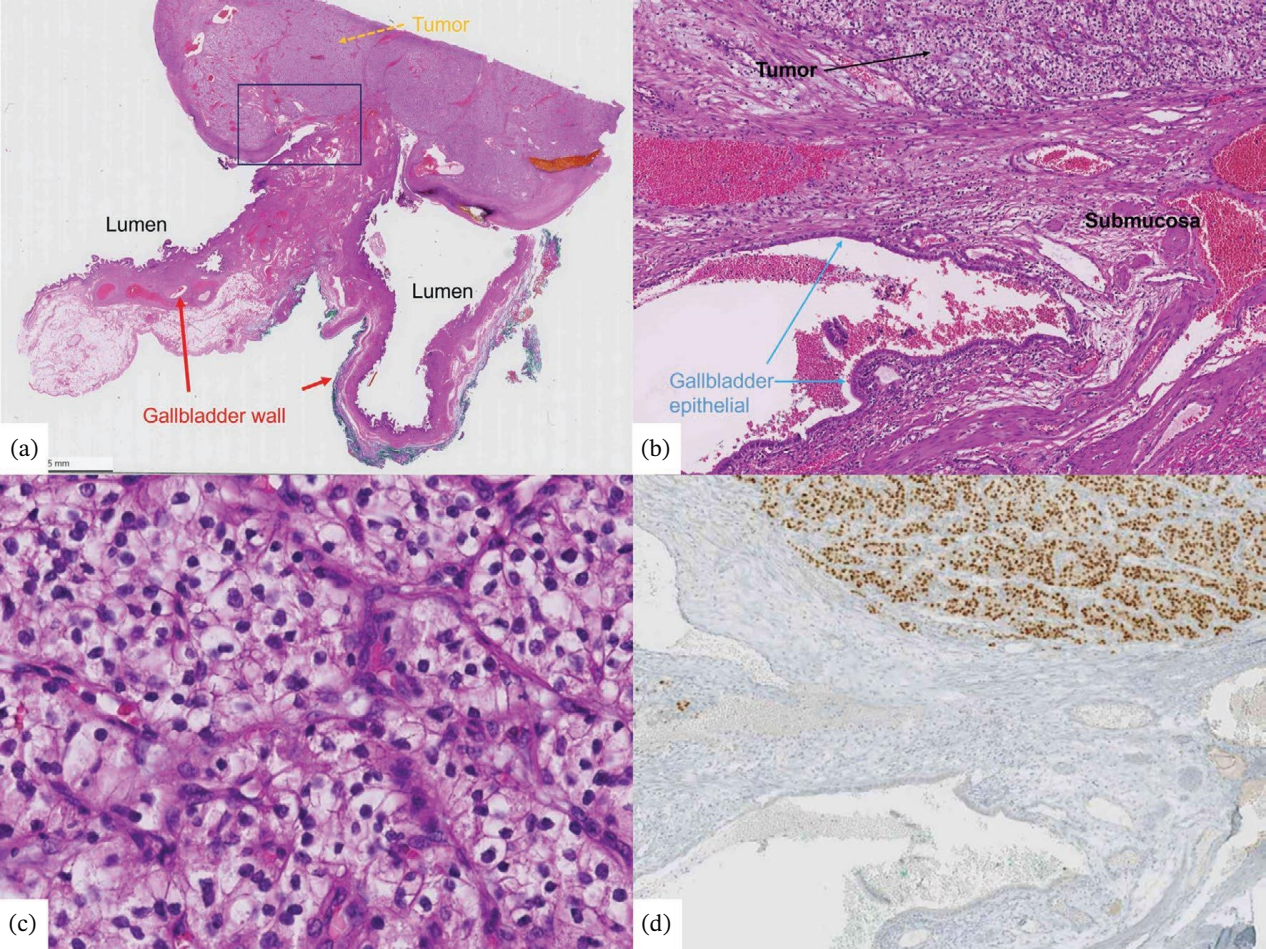
Histological analysis—(a) Overview; (b) enlarged section of A containing the tumor; (c) tumor: consists of light (whitish), relatively pale cells, already morphologically well suited to RCC; and (d) immunohistochemistry: tumor positive for PAX8 (same section as B).

Two weeks later, the patient underwent laparoscopic cholecystectomy. Histological analysis confirmed that the lesion was a metastasis of ccRCC (Figure 8). The surgical procedure, known as resection, was performed with healthy margins.

A subsequent echo‐endoscopy with biopsy of the pancreatic lesion was performed 4 days after the cholecystectomy. However, the histological examination yielded no significant findings due to the poor quality of the specimen. A subsequent biopsy was deemed unnecessary, as the gallbladder lesion had been definitively diagnosed as ccRCC metastasis.

The patient′s case was subsequently discussed in a multidisciplinary tumor board. Considering her age and the risk of intraoperative morbidity associated with pancreatic surgery, a watchful waiting strategy including a follow‐up CT scan at 3 months was recommended. This revealed the progression of the pancreatic lesion, as well as the appearance of a new lesion suspicious of metastasis in the head of the pancreas.

In a multidisciplinary conference, radiotherapy administered to the lesions was recommended and initiated targeting both metastases at 11 months postnephrectomy (August 2023). In a postradiotherapy follow‐up CT, regression of both lesions was noted. However, subsequent imaging revealed the presence of three new pancreatic metastases. The latter demonstrated stability on subsequent follow‐up examinations, thereby guiding the therapeutic strategy toward active surveillance.

In November 2024, significant progression of certain lesions was noted, leading to the initiation of combined PD‐1 and CTLA‐4 blockade with nivolumab and Ipilimumab, respectively, according to the CHECKMATE‐214 trial [7]. In March 2025, the patient developed immuno‐induced hypophysitis, resulting in cortisol deficiency that was effectively treated with hydrocortisone, with rapid improvement in her general condition.

Following complete recovery from hypophysitis and in accordance with guideline recommendations, nivolumab was resumed as maintenance therapy in the context of stable disease. In May 2025, as the disease remained under control, without any major progression, and the treatment was well tolerated, the immunotherapy was continued with an administration interval extended to 4 weeks.

Patient characteristics are summarized in Table 1.

**Table 1:** Summary of different cases.

Criteria	Case 1	Case 2	Case 3	Case 4
Age/gender	77 years/female	76 years/female	46 years/male	70 years/female
Tumor characteristics/TNM classification at the time of nephrectomy	Clear cell renal cell carcinoma (pT3b pN0 R0, Fuhrman II)	Clear cell renal cell carcinoma (pT3a N0, Fuhrman I)	Clear cell renal cell carcinoma (nephrectomy history)	Clear cell renal cell carcinoma (pT2a N0 R0)
Metastatic site	Pancreas	Thyroid and Pancreas	Nasal cavity, pancreas, liver	Pancreas, gallbladder
Clinical status at diagnosis	Asymptomatic	Asymptomatic	Epistaxis	Macrohaematuria (due to primary tumor)
Time from nephrectomy to onset of metastases	3 years	Synchronous	15 years	Synchronous
Treatment	Active Surveillance and after Immunotherapy because of progress	Thyroid lobectomy and active monitoring	Endonasal resection, immunotherapy (pembrolizumab/axitinib) and excision of each metastatic lesion	Cholecystectomy, radiotherapy, and immunotherapy
Evolution	Slow growth of metastases over time. Growth of a new lesion in the body of the pancreas leading to compression of the digestive tract	Stable metastases	Progression of the liver metastasis	Appearance of new, stable metastases
Alive/deceased	Still alive	Deceased	Still alive	Still alive

## 3. Discussion

ccRCC is characterized by a marked propensity for metastatic spread, which may involve a wide range of organs. One of the characteristics of these metastases is that they can remain asymptomatic for a long time, thus leading to a diagnostic delay. Some are discovered during the staging workup of the primary tumor, whereas others are discovered during follow‐up. Although CT scans with contrast injection are the gold standard for both detection and monitoring, MRI represents an alternative for patients with contraindications to contrast injection [8–10]. Due to its low sensitivity and specificity, FDG‐PET/CT is not used routinely but only in certain advanced stages [11].

Recent advances in molecular imaging have led to the development of CAIX‐targeted PET tracers, including radiolabeled antibodies such as girentuximab and small‐molecule ligands like DPI‐4452 and NY104. Furthermore, the long physical half‐life of ^89^Zr (78.4 h) requires delayed imaging of nearly 5 days and raises radiation exposure concerns, limiting clinical practicality [12–16].

Confirmation of metastatic disease is achieved through histopathological analysis of biopsy or surgical specimens, ideally correlated with the primary tumor [17, 18]. Indeed, these metastases, as well as the tumor, exhibit the same immunohistochemical characteristics and express certain specific proteins such as CAIX, PAX8, CD10, and vimentin [19, 20].

Treatment decisions in metastatic ccRCC rely on international guidelines but must be adapted to individual patient profiles and prognostic risk stratification, including the IMDC score. The two types of molecules used when systemic treatment is recommended are ICI and tyrosine kinase inhibitors targeting VEGFR (VEGFR‐TKI), administered according to well‐established protocols [21–23].

Pancreatic metastases from ccRCC are rare and can remain asymptomatic for a long time, thus progressing indolently. When suspected, an ultrasound‐guided biopsy should be considered in some cases for confirmation. Treatment is exclusively systemic [18].

Even rarer, metastases to the nasal cavity and paranasal sinuses should be suspected in patients presenting with symptoms such as nasal obstruction, epistaxis, or facial pain, as delayed diagnosis makes management difficult. In addition to systemic therapy, resection is important in well‐selected cases [24, 25].

Gallbladder metastases are rare, and although the majority of cases are asymptomatic, some can often present with gastrointestinal symptoms mimicking cholecystitis. When the diagnosis is established, complete resection (R0) should be performed [26, 27].

Also rare, metastases of ccRCC to the thyroid gland are, like the previous ones, mostly asymptomatic, but in a minority, they can present with symptoms of dyspnea, dysphonia, and a cervical mass without thyroid dysfunction. The prognosis is generally favorable in cases of isolated metastasis and when total thyroidectomy is performed [28, 29].

## 4. Conclusion

The present series of four cases illustrates the unusual and rare metastatic sites of ccRCC, particularly in the pancreas, nasal cavity, gallbladder, and thyroid. Often asymptomatic, these lesions are frequently discovered incidentally, sometimes several years after the diagnosis of the primary tumor. It is therefore crucial to maintain surveillance for patients diagnosed with ccRCC, even if no symptoms are reported, because early detection and management through a multidisciplinary strategy lead to a better prognosis than when these lesions are detected late.

## Author Contributions

**Roosvelt Tessa Djambong:** conception, organization, methodology, visualization, writing—original draft, writing—review and editing. **Bril Laurel Follah Feudjio:** conception, organization, methodology, visualization, writing—original draft, writing—review and editing. **Stéphane Rysselinck:** methodology, writing—original draft, writing—review and editing. **Emmanuel Seront:** writing—review and editing. **Günter Niegisch:** methodology, supervision, writing—original draft, writing—review and editing. **Christina Neppl:** methodology, writing – original draft. **Alexander Müller:** conception, methodology, supervision, writing—original draft, writing—review and editing. Roosvelt Tessa Djambong and Bril Laurel Follah Feudjio contributed to the work equally and should be regarded as co‐first authors.

## Funding

No funding was received for this manuscript.

## Disclosure

All authors have read and approved the final version of the manuscript; Roosvelt Tessa Djambong had full access to all of the data in this study and takes complete responsibility for the integrity of the data and the accuracy of the data analysis.

## Consent

No written consent has been obtained from the patients, as there is no patient identifiable data included in this case series.

## Conflicts of Interest

The authors declare no conflicts of interest.

## Data Availability

The data that support the findings of this study are available from the corresponding author upon reasonable request.
